# Supplementary material for: Remodelling of tumour microenvironment by microwave ablation potentiates immunotherapy of AXL-specific CAR T cells against non-small cell lung cancer
Source: Nat Commun. 2022 Oct 19;13:6203. doi: 10.1038/s41467-022-33968-5 (PMC9581911; doi:10.1038/s41467-022-33968-5)
Supplement: Supplementary file 1 — Supplementary Information [file 41467_2022_33968_MOESM1_ESM.pdf]

Remodelling of tumour microenvironment by microwave ablation potentiates immunotherapy of AXL-specific CAR T cells against non-small cell lung cancer

Supplementary Information

SUPPLEMENTARY FIGURES

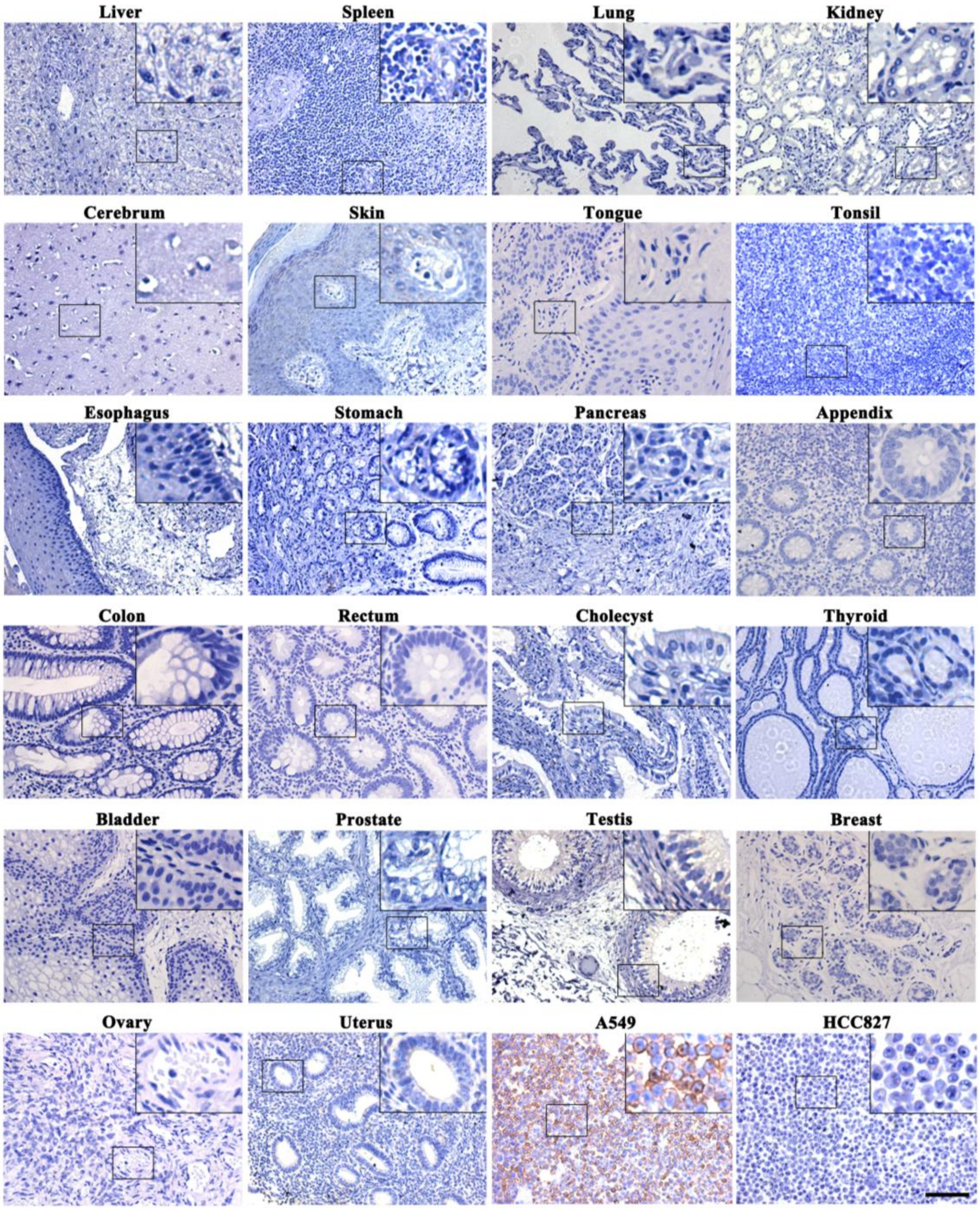

Supplementary Fig. 1 AXL expression in human normal tissues. Twenty-two different human normal tissue samples were immunostained with an anti-AXL antibody to determine AXL expression level. Representative staining images (magnification × 400) are shown. Scale bar represents 100 μm. Paraffin-embedded A549 and HCC827 cells served as positive and negative control, respectively. Data summarize one independent experiments.

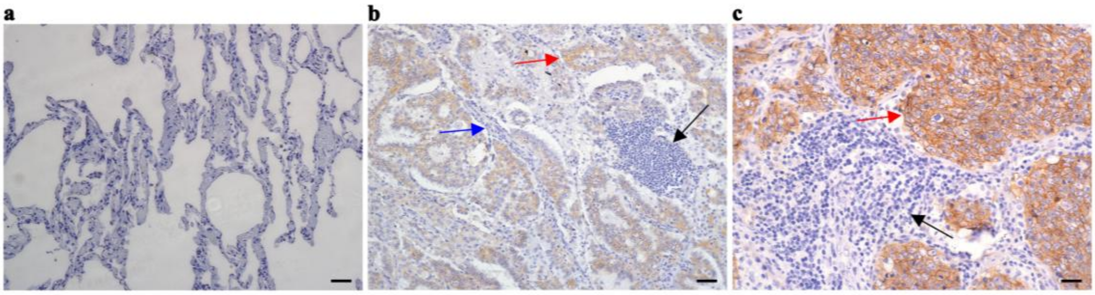

Supplementary Fig. 2 AXL expression in human cancerous and adjacent non-tumour lung tissues. AXL detection in NSCLC cancerous (**b/c**, **red arrow**) and adjacent non-tumour lung tissues, including areas with non-malignant tissue (**a**), vascular cells (**b**, **blue arrow**) and immune infiltrates (**b/c**, **black arrow**). Representative staining images (magnification × 400) are shown (n=5). Scale bars represent 50 μm.

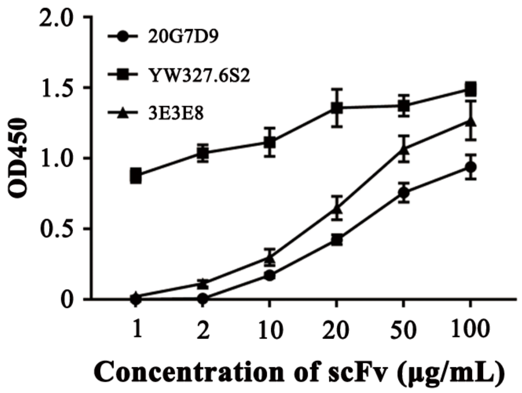

Supplementary Fig. 3 AXL-specific affinity was examined via ELISA. Data were obtained from three independent experiments and expressed as mean ± SD. Source data are provided as a Source Data file.

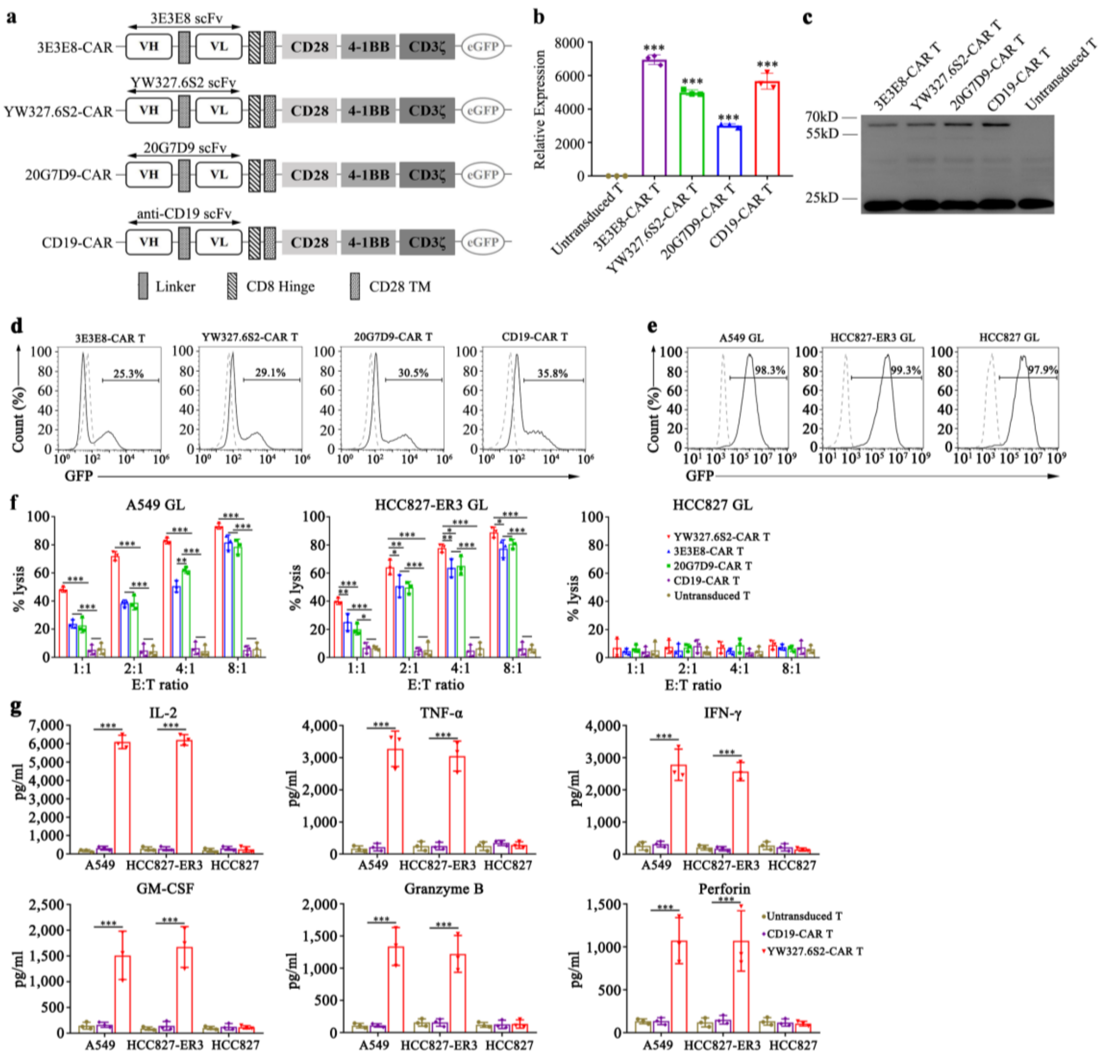

Supplementary Fig. 4 Generation of CAR T cells and their anti-cancer cytotoxicity assay in vitro. **a**, Schematic diagram of the AXL- and CD19-CAR transgene domains. **b**, Relative expressions of different CAR mRNAs normalized to GAPDH in CAR T cells were detected by qPCR. **c**, Western blotting analysis of CAR expression in transduced AXL- and CD19-CAR T cells. A CD3 ζ-specific antibody was used to detect endogenous and chimeric CD3 ζ. One of three repetitions with similar results is shown. **d**, Percentage of AXL- and CD19-CAR transduced CAR T cells detected by flow cytometry. GFP served as a marker of CAR expression. Dash line indicated isotype control. **e**, GFP and Luciferase (GL) expression was detected in A549 GL, HCC827-ER3 GL, and HCC827 GL cells by flow cytometry. GFP served as a marker of luciferase

expression. **f**, Antitumour cytotoxicity assay of AXL-CAR T cells. The effector T cells were co-cultured for 24 hrs with target cells (1×10^4) at effector (E): target (T) ratios of 1:1, 2:1, 4:1, and 8:1 in a total volume of 100 μL. **g**, Detection of IL-2, TNF-α, IFN-γ, GM-CSF, Granzyme B, and Perforin secretion by effector cells after coculture with target cells for 24 hrs at an E:T ratio of 1:1. Data are expressed as mean ± SD of triplicate samples from three separate experiments and analysed by one-way ANOVA with Tukey's multiple comparisons test. *$p < 0.05$, **$p < 0.01$, ***$p < 0.001$. Source data and exact *p* values are provided as a Source Data file.

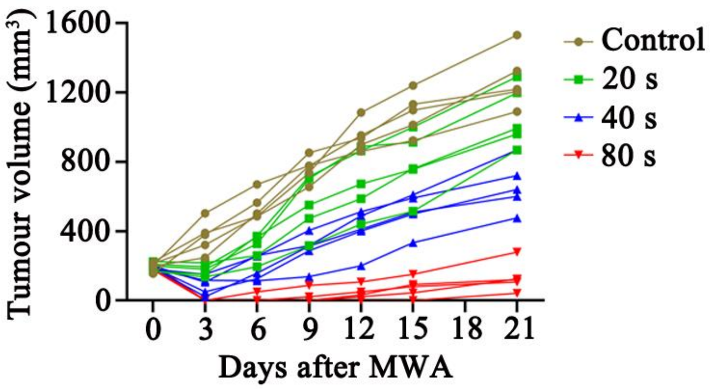

Supplementary Fig. 5 Power-time gradient ablation. Tumour volume curve after abltion with different ablation parameters. Tumour volume = (length × width^2)/2. Data summarize one independent experiment (n = 5 mice per group). Source data are provided as a Source Data file.

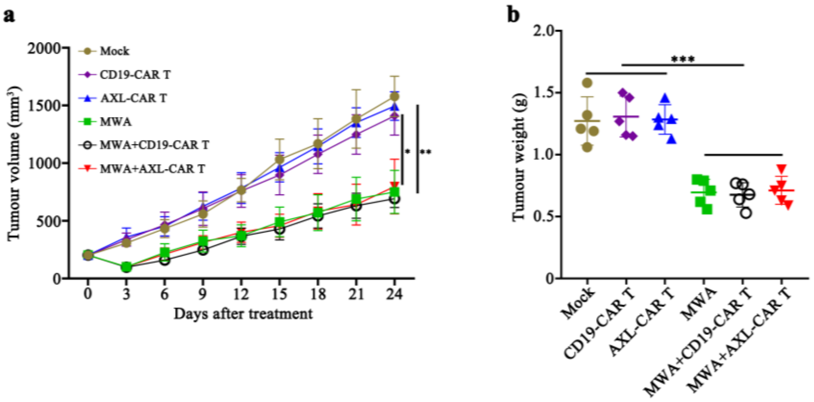

Supplementary Fig. 6 Combination of MWA and CAR T cells against HCC827-derived CDX tumour. a.NSG mice received a subcutaneous injection of 2×10^6 HCC827 cells. When tumour volume reached about 200 mm^3, mock, MWA +/- 1×10^7 CD19- or AXL-CAR T cells (i.v.) were administrated against HCC827-bearing tumour. Tumour volume was measured every three days (Mock vs MWA $p = 0.0008$, Mock vs MWA+CD19-CAR T $p = 0.0006$, Mock vs MWA+AXL-CAR T $p = 0.004$, CD19-CAR T vs MWA $p = 0.003$, CD19-CAR T vs MWA+CD19-CAR T $p = 0.001$, CD19-CAR T vs MWA+AXL-CAR T $p = 0.02$, AXL-CAR T vs MWA $p = 0.001$, AXL-CAR T vs MWA+CD19-CAR T $p < 0.0001$, AXL-CAR T vs MWA+AXL-CAR T $p = 0.008$, two-way ANOVA with Tukey's multiple comparisons test). **b**. Tumour weight was calculated at the end point (Mock/CD19-CAR T/AXL-CAR T vs MWA/MWA+CD19-CAR T/MWA+AXL-CAR T $p < 0.0001$, one-way ANOVA with Tukey's multiple comparisons test). Data are presented as mean ± SD (**a, b,** n = 5 mice per group). $^*p < 0.05$, $^{**}p < 0.01$, $^{***}p < 0.001$. Source data are provided as a Source Data file.

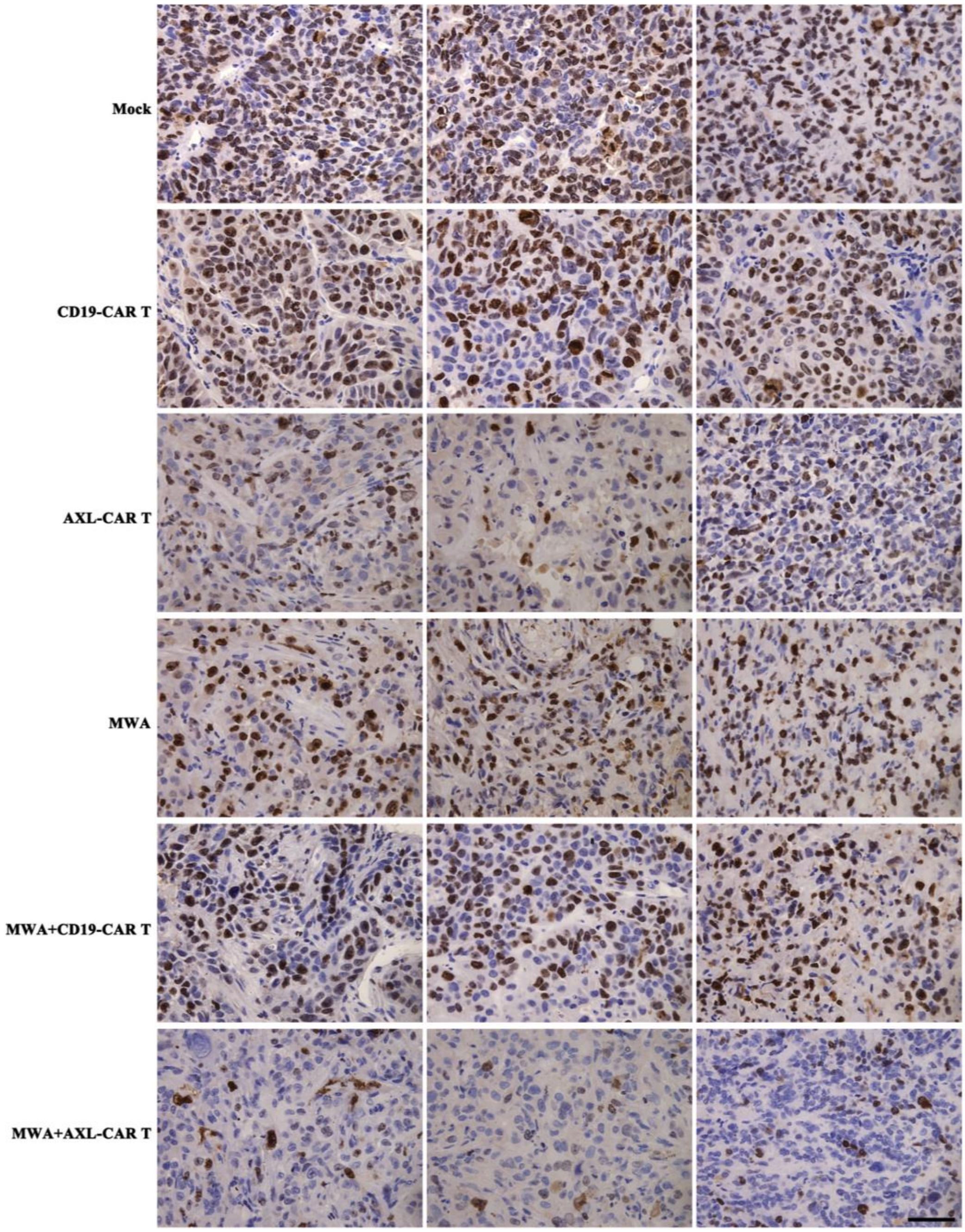

Supplementary Fig. 7 Ki-67 expression in tumours removed from mice of different groups in figure 3. Representative staining image fields (magnification ×400) are shown (n = 3 per group). Scale bar, 50 μm. Data summarize one independent experiment.

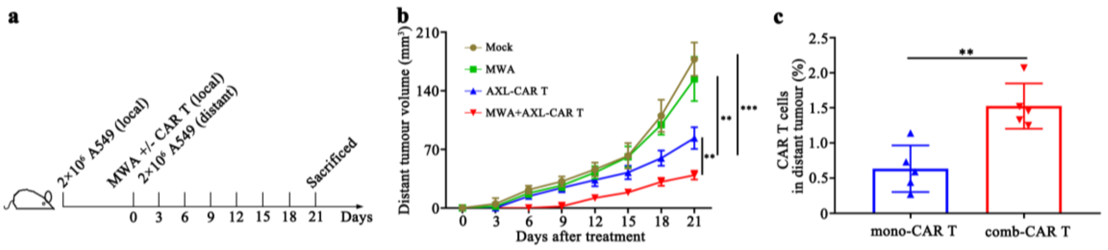

Supplementary Fig. 8 Antitumour efficacy of MWA +/- AXL-CAR T cells against A549-derived distant tumour. a. Schematic representation of the experiment. Briefly, NSG mice received a subcutaneous injection of 2×10^6 A549 cells in the left flank (local). When tumour volume reached about 200 mm^3 (day 0), mock, MWA +/- AXL-CAR T cells (i.v.) were administrated against A549-bearing tumour. At the same day (day 0), another 2×10^6 A549 cells were inoculated to the right flank (distant). **b**. Tumour volume in the distant flank (Mock vs AXL-CAR T/MWA+AXL-CAR T p = 0.0002, MWA vs AXL-CAR T p = 0.008, MWA vs MWA+AXL-CAR T p = 0.002, AXL-CAR T vs MWA+AXL-CAR T p = 0.003, two-way ANOVA with Tukey's multiple comparisons test). **c.** Percentages of CAR T cells (GFP^+) in tumour by flow cytometry (mono-CAR T vs comb-CAR T p = 0.003, two-sided unpaired t-test). Data are presented as the mean ± SD of n = 5 mice per group (**b**, **c**). *$p < 0.05$, **$p < 0.01$, ***$p < 0.001$. Source data are provided as a Source Data file.

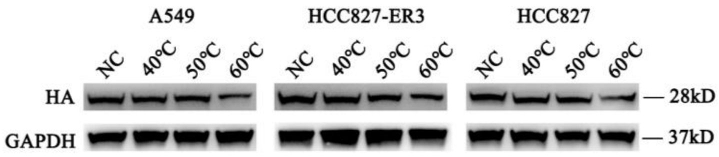

Supplementary Fig. 9 Hyaluronic acid expression of lung cancer cell lines under different temperature by WB. A549, HCC827-ER3, HCC827 were uncubated in 40, 50 or 60 ℃ for 5 min. Proteins from these cells were harvested in 24 hours for WB detection by staining anti-human HA primary antibody. One of three repetitions with similar results is shown. Source data are provided as a Source Data file.

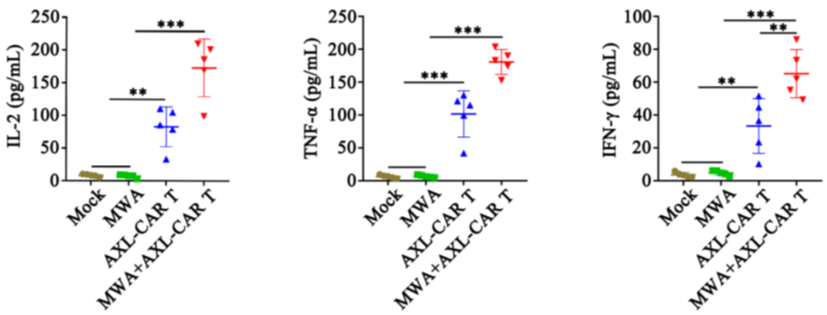

Supplementary Fig. 10 Cytokine levels in plasma of individual mice obtained from blood on day 42 after treatment in PDX bearing mice. Data reflected the mean ± SD of each group n = 5. (IL-2: Mock/MWA vs AXL-CAR T $p = 0.002$, Mock/MWA vs MWA+AXL-CAR T $p < 0.0001$, AXL-CAR T vs MWA+AXL-CAR T $p = 0.0004$. TNF-α: Mock/MWA vs AXL-CAR T $p < 0.0001$, Mock/MWA/AXL-CAR T vs MWA+AXL-CAR T $p < 0.0001$. IFN-γ: Mock vs AXL-CAR T $p = 0.003$, MWA vs AXL-CAR T $p = 0.005$, AXL-CAR T vs MWA+AXL-CAR T $p = 0.002$, Mock/MWA vs MWA+AXL-CAR T $p < 0.0001$. one-way ANNOVA with Tukey's multiple comparisons test). *$p < 0.05$, **$p < 0.01$, ***$p < 0.001$. Source data are provided as a Source Data file.

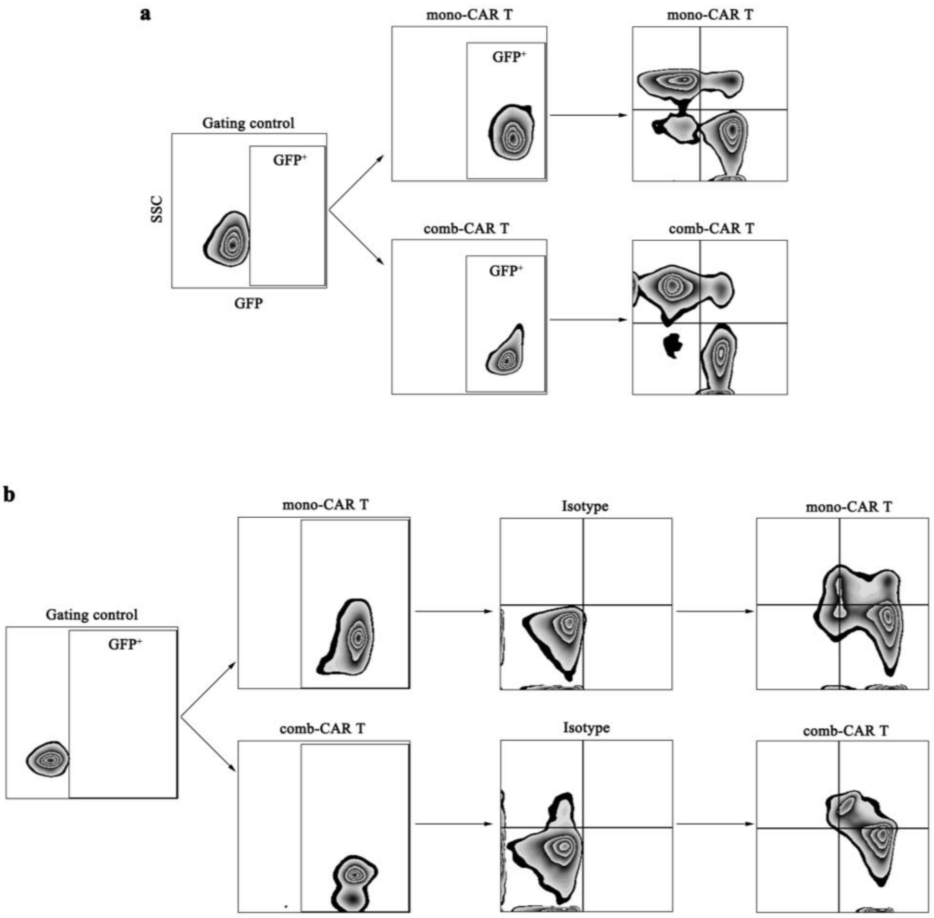

Supplementary Fig. 11 Exemplifying of the gating strategy in figure 4g (a) and figure 6c/d (b).

SUPPLEMENTARY TABLES

Human normal tissue	AXL expression (cases)				Total
	0	1+	2++	3+++	
Liver	20	0	0	0	20
Spleen	10	0	0	0	10
Lung	24	0	0	0	24
Kidney	10	0	0	0	10
Cerebrum	10	0	0	0	10
Esophagus	3	0	0	0	3
Stomach	8	0	0	0	8
Colon	17	1	0	0	18
Rectum	18	1	0	0	19
Appendix	13	0	0	0	13
Gall bladder	13	0	0	0	13
Tongue	3	0	0	0	3
Tonsil	6	0	0	0	6
Thyroid	5	0	0	0	5
Pancreas	5	0	0	0	5
Bladder	3	0	0	0	3
Prostate	5	0	0	0	5
Testis	3	0	0	0	3
Breast	12	0	0	0	12
Ovary	5	0	0	0	5
Uterus	5	0	0	0	5
Skin	8	0	0	0	8
**Total**	206 (99%)	2 (1%)	0	0	208 (100%)

Supplementary Table 1. AXL expression in human normal tissues. Twenty-two kinds of human normal tissue (varied from 3 to 24 cases) collected, AXL expression was detected via IHC in **Supplementary Fig. 1**.

	EGFR TKI-resistant NSCLC	EGFR-WT NSCLC
Tumour type		
ACC	22 (55.0%)	31 (62.0%)
SqCC	14 (35.0%)	14 (28.0%)
NSC	4 (10.0%)	5 (10.0%)
AXL expression		
negative	10 (25.0%)	18 (36.0%)
positive	30 (75.0%)	32 (64.0%)
1+	15 (37.5%)	17 (34.0%)
2++	7 (17.5%)	9 (18.0%)
3+++	8 (20.0%)	6 (12.0%)
Total	40 (100%)	50 (100%)

Supplementary Table 2. Histologic diagnoses and clinical characteristics of 90 NSCLC patients in Fig. 1a.

ID	Tumour type	EGFR alteration	TKI
1	ACC	Del19	Erlotinib
2	ACC	Del19	Erlotinib
3	SqCC	Del19	Gefitinib
4	ACC	L858R	Erlotinib
5	ACC	Del19	Erlotinib
6	SqCC	Del19	Erlotinib
7	ACC	Del19	Gefitinib
8	SqCC	L858R	Gefitinib

Supplementary Table 3. Clinical characteristics of eight paired EGFR-resistant NSCLC tissues in Fig. 1b.

	AST (U/L)	ALT (U/L)	BUN (mg/dL)	TBIL (μmol/L)
Mock	133.2±16.0	44.8±11.6	21.5±3.0	20.4±3.5
CD19-CAR T	135.8±19.1	44.0±12.7	22.1±3.1	18.8±2.8
AXL-CAR T	133.8±22.7	47.2±11.0	22.5±3.9	21.9±3.0
MWA	137.3±16.0	46.4±12.8	22.0±3.4	20.9±2.7
MWA+CD19-CAR T	134.4±24.3	48.1±11.7	22.1±3.3	21.0±5.1
MWA+AXL-CAR T	135.2±15.6	46.5±9.6	21.4±3.2	19.8±2.9

Supplementary Table 4. Serum biochemical analyses in different group. AST: aspertate aminotransferase; ALT: alanine transaminase; BUN: blood urea nitrogen; TBIL: total bilirubin. Source data are provided as a Source Data file.

SUPPLEMENTARY METHODS

Expression and affinity verification of anti-AXL scFvs

As described in previous studies[1], YW327.6S2, 3E3E8, and 20G7D9 bound specifically to extracellular AXL. The heavy and light chain gene sequences of the three anti-AXL antibodies YW327.6S2, 3E3E8, and 20G7D9 were cloned into the mammalian expression vector. First, 20 μL of plasmids (1 μg/mL) and 40 μL of PEI transfection reagents (1 μg/mL) were mixed in 2 mL of endotoxin-free PBS and incubated for 15 min at room temperature (RT). Then the mixture was added dropwise to FreeStyle 293-F cells in 30 mL of FreeStyle 293 expression culture medium. Four days post-transfection, the culture supernatant was harvested. Antibodies were purified by the Protein A Sepharose TM chromatography (GE Healthcare). The protein purity was detected by SDS-PAGE in a 12% gel.

ELISA was then performed to verify the AXL-specific affinity. The AXL antigen (R&D system) was coated on the 96-well ELISA plates (Nunc) at 4 ℃ overnight. The wells were blocked with PBS containing 4% non-fat milk for 1 h at RT. AXL protein was bound with serial dilutions of anti-AXL scFvs at RT for 1 h, followed by the detection by a horseradish peroxidase-conjugated anti-Flag tag antibody (Sigma) as the secondary antibody for 1 h at RT. 3,3′,5,5′-Tetramethylbenzidine (Beyotime) was added as substrate, and the reaction was read at 450 nm (Supplementary Fig. 3).

CAR design and lentivirus production

To generate CARs targeting AXL, the genes of three anti-AXL scFv[2-5] and anti-CD19

(control scFv), followed by CD8 leader, CD8 hinge, CD28 transmembrane, and composite CD28-4-1BB-CD3ζ intracellular signalling domains under the control of the EF-1α promoter were codon optimised and synthesised by Genscript Co. Ltd. (Nanjing, China) and then subcloned into the backbone lentiviral vector pWPXLd-2A-eGFP (Supplementary Fig. 4a). The sequence of each cloned CAR was verified by sequencing.

Lentivirus particles were produced in HEK-293T cells after polyethyleneimine (Sigma-Aldrich)-mediated transfection with the constructed vector, together with two auxiliary packaging plasmids, psPAX2 and pMD.2G. Supernatants were harvested at 48 and 72 h post-transfection and filtered through a 0.45-μm filter (Millipore) to remove cell debris.

Isolation, transduction, and expansion of primary human T lymphocytes

Peripheral blood mononuclear cells (PBMCs) were isolated from buffy coats of healthy donors using Lymphoprep (Stemcell). T cells were positively selected from PBMCs using the MACS Pan T Cell Isolation Kit (Miltenyi Biotec) and activated using microbeads coated with anti-human CD3/CD28 (Miltenyi Biotec) at a 1:1 bead:cell ratio for 24-72 h in the T cell medium (Miltenyi Biotec). Sorted fresh T cells were stained with anti-human CD3-FITC (clone UCHT1). Transduction was performed as previously described[6]. Transduction efficiency and CAR expression were assessed after 72 h by the percentage of GFP^+ cells determined by flow cytometry, qPCR and western blotting (Supplementary Fig. 4b–d). Informed consents

from healthy PBMC donors were obtained, and all procedures were approved by the Institutional Review Board of The Second Affiliated Hospital of the Guangzhou Medical University.

Quantitative real-time polymerase chain reaction (qPCR)

The expression levels of different candidate mRNAs was detected by qPCR as described previously[7] on an Applied Biosystems instrument. Expression level of the glyceraldehyde-3-phosphate dehydrogenase (*GAPDH*) gene was used as endogenous control (Supplementary Fig. 4b). All probes were obtained from Applied Biosystems except the primers for the following genes:

GAPDH - sense: 5′-GCACCGTCAAGGCTGAGAAC-3′

GAPDH - antisense: 5′-TGGTGAAGACGCCAGTGGA-3′

scFv of 3E3E8 - sense: 5′-GCACAGCAACGGCAACACCTA-3′

scFv of 3E3E8 - antisense: 5′-CCGCTAAACCTATCGGGCACT-3′

scFv of YW327.6S2 - sense: 5′-GCAGCATCTGGCTTTTCTC-3′

scFv of YW327.6S2 - antisense: 5′-TTCACGGAGTCGGCATAGT-3′

scFv of 20G7D9 - sense: 5′-TACAATGAGAAGTTCAACGACCG-3′

scFv of 20G7D9 - antisense: 5′-GTCCCCAGTATGCGAACCAG-3′

scFv of CD19 - sense: 5′-ACTACATCCTCCCTGTCTGCC-3′

scFv of CD19 - antisense: 5′-CCACTGCCACTGAACCTTGA-3′

***In vitro* cytotoxicity verification**

A549 GL, HCC827 GL, and HCC827-ER3 GL target cells were incubated with Mock, CD19- or AXL-CAR T cells at the effector-to-target (E:T) ratios of 8:1, 4:1, 2:1, and 1:1, respectively, in triplicate wells of U-bottomed 96-well plates at 37 ℃. Target cell viability was monitored 24 h later by adding 150 μg/mL D-luciferin (Cayman Chemical) at 100 μL/well. Then, the viability percentage (%) was calculated as follows: experimental signal/maximal signal × 100. The percentage of specific cell lysis was calculated as follows: 100% − viability percentage (Supplementary Fig. 4f).

Cytokine release assay

Target cells (1×10^4) were incubated with effector cells (1×10^4) in U-bottomed 96-well plates for 24 h. The culture supernatants were then collected and analysed for the secretion of IL-2, TNF-α, IFN-γ, GM-CSF, granzyme B, and perforin using ELISA kits (R&D Systems) according to the manufacturer's protocol (Supplementary Fig. 4g).

Power-time gradient ablation assay

The microwave ablation instrument can use different power and time combinations to achieve the best ablation effect. In order to achieve partial ablation, we have optimised the parameters via a gradient ablation experiment to faithfully mimic the tumour residue or marginal recurrence after ablation. Our experiments started when subcutaneous HCC827-ER3 cell-derived tumours in NSG mice reached a volume of 200 mm^3. Based on the manufacturer's ablation parameters, the tumour-bearing mice

were divided into four groups: control; 10 W, 20 s; 10 W, 40 s; and 10 W, 80 s. Tumour volume was calculated after the treatment to assess the efficiency of tumour burden reduction (Supplementary Fig. 5).

Combination strategy exhibits superior tumour suppression in A549-derived distant tumour

To address how MWA can enhance antitumour efficacy of CAR T cells by modulating TMEs, we established one local (left flank) and distant (right flank) tumour model by inoculating A549 cells in NSG mice. When the local tumour volume reached approximately 200 mm^3, they were allocated to the following groups: A. mock, B. MWA, C. AXL-CAR T (i.v.), D. MWA+AXL-CAR T (i.v.). A549 cells were inoculated in the right flank (distant) at the same day. The tumour growth and CAR T cell infiltration in distant tumour were observed (Supplementary Fig. 8).

***In vitro* gradient temperature assay**

To examine whether high temperature affects the expression of hyaluronic acid in lung cancer cell lines, we performed the gradient temperature assay. A549, HCC827 and HCC827-ER3 cells were plated on day 0 and when cell density in the dish reached about 30%, these cells were incubated in 40 ℃, 50 ℃, or 60 ℃ for 5 min, to mimic the ablation temperature. Proteins from these cells were harvested in 24 h for western blotting (Supplementary Fig. 9).

SUPPLEMENTARY REFERENCES

1. Liu, J., et al. Targeting B7-H3 via chimeric antigen receptor T cells and bispecific killer cell engagers augments antitumor response of cytotoxic lymphocytes. *Journal of hematology & oncology*. **14**, 21-21 (2021).

2. Leconet, W., et al. Preclinical validation of AXL receptor as a target for antibody-based pancreatic cancer immunotherapy. *Oncogene*. **33**, 5405-14 (2014).

3. Ye, X., et al. An anti-Axl monoclonal antibody attenuates xenograft tumor growth and enhances the effect of multiple anticancer therapies. *Oncogene*. **29**, 5254-64 (2010).

4. Pei, L., et al. Anti-Axl antibodies and methods of use. *United States patent US 8853369 B2* (2012).

5. Robert, B., et al. Anti-Axl antibodies and uses thereof. *United States patent US 20140302041 A1* (2012).

6. Lv, J., et al. Mesothelin is a target of chimeric antigen receptor T cells for treating gastric cancer. *Journal of hematology & oncology*. **12**, 18 (2019).

7. Cao, B., et al. Development of mesothelin-specific CAR NK-92 cells for the treatment of gastric cancer. *International Journal of Biological Sciences*. **17**, 3850-3861 (2021).
