# Supplementary material for: Remodelling of tumour microenvironment by microwave ablation potentiates immunotherapy of AXL-specific CAR T cells against non-small cell lung cancer
Source: Nat Commun. 2022 Oct 19;13:6203. doi: 10.1038/s41467-022-33968-5 (PMC9581911; doi:10.1038/s41467-022-33968-5)
Supplement: Supplementary file 3 — Reporting Summary [file 41467_2022_33968_MOESM3_ESM.pdf]

nature portfolio

Corresponding author(s): Qi Zhao, Zhenfeng Zhang

Last updated by author(s): Oct 5, 2022

Reporting Summary

Nature Portfolio wishes to improve the reproducibility of the work that we publish. This form provides structure for consistency and transparency in reporting. For further information on Nature Portfolio policies, see our Editorial Policies and the Editorial Policy Checklist.

Statistics

For all statistical analyses, confirm that the following items are present in the figure legend, table legend, main text, or Methods section.

n/a	Confirmed	
☐	☒	The exact sample size (*n*) for each experimental group/condition, given as a discrete number and unit of measurement
☐	☒	A statement on whether measurements were taken from distinct samples or whether the same sample was measured repeatedly
☐	☒	The statistical test(s) used AND whether they are one- or two-sided<br>*Only common tests should be described solely by name; describe more complex techniques in the Methods section.*
☒	☐	A description of all covariates tested
☒	☐	A description of any assumptions or corrections, such as tests of normality and adjustment for multiple comparisons
☐	☒	A full description of the statistical parameters including central tendency (e.g. means) or other basic estimates (e.g. regression coefficient) AND variation (e.g. standard deviation) or associated estimates of uncertainty (e.g. confidence intervals)
☐	☒	For null hypothesis testing, the test statistic (e.g. *F*, *t*, *r*) with confidence intervals, effect sizes, degrees of freedom and *P* value noted<br>*Give P values as exact values whenever suitable.*
☒	☐	For Bayesian analysis, information on the choice of priors and Markov chain Monte Carlo settings
☒	☐	For hierarchical and complex designs, identification of the appropriate level for tests and full reporting of outcomes
☒	☐	Estimates of effect sizes (e.g. Cohen's *d*, Pearson's *r*), indicating how they were calculated

Our web collection on statistics for biologists contains articles on many of the points above.

Software and code

Policy information about availability of computer code

Data collection	1.For Flow Cytometry, data collection was performed by using a NovoCyteTM flow cytometer (ACEA Novocyte D2060R).<br>2.For subcutaneous tumour microwave ablation, procedure was performed by using a commercial MWA modality (Vision-China Medical Devices R&D center) without software.<br>3.For PO2, data was detected by using a polarographic electrode (POG-203; Unique Medical).<br>4.For tumour blood flow rate monitor, data collection was achieved by using the laser speckle equipment (MoorFLPI-2, Moor Instruments).<br>5.For Bioluminescence imaging, data was collected by a living Imaging System (IVIS 100 Series).<br>6.ECAR and OCR were measured using a XFe96 Extracellular Flux Analyser (Seahorse Bioscience) with the Seahorse Cell Energy Phenotype Test Kit.<br>7.Immunofluorescence and immunohistochemistry examined with a fluorescence microscope (Olympus IX53).<br>8.For mitochondrion detection, images were acquired with a transmission electron microscope (FEI Tecnai G2 Spirit Biotwin, Eindhoven, the Netherlands).
Data analysis	1.Statistical analyses were performed with GraphPad Prism 8.0.<br>2.Flow cytometry was performed with FlowJO V10.<br>3.Grey-scale analysis of WB images was performed by ImageJ (V1.8.0).<br>4.Average emissions were quantified using Living Image software (LI4.5.5, Xenogen).

For manuscripts utilizing custom algorithms or software that are central to the research but not yet described in published literature, software must be made available to editors and reviewers. We strongly encourage code deposition in a community repository (e.g. GitHub). See the Nature Portfolio guidelines for submitting code & software for further information.

Data

Policy information about availability of data

All manuscripts must include a data availability statement. This statement should provide the following information, where applicable:

- Accession codes, unique identifiers, or web links for publicly available datasets
- A description of any restrictions on data availability
- For clinical datasets or third party data, please ensure that the statement adheres to our policy

All the data are available within the Article, Supplementary Information or Source Data file. Source data are provided as a Source Data file.

Field-specific reporting

Please select the one below that is the best fit for your research. If you are not sure, read the appropriate sections before making your selection.

☒ Life sciences ☐ Behavioural & social sciences ☐ Ecological, evolutionary & environmental sciences

For a reference copy of the document with all sections, see nature.com/documents/nr-reporting-summary-flat.pdf

Life sciences study design

All studies must disclose on these points even when the disclosure is negative.

Sample size	Sample sizes of the patients were based on the biopsy or surgery paraffin section. For in vitro experiment, cells >=100000 for flow-cytometry; for in vivo study, 1×10^6 for pulmonary matastasis model, 2×10^6 for subcutaneous tumour model, or approximately 5mm*5mm*mm tumour samples were used for PDX tumour model construction; and each experiment in vitro repeated three times and mouse n>=5 was used in vivo experiment.
Data exclusions	No data were excluded, and all samples were included in data analysis.
Replication	All experiments were experimental duplicates or triplicates or independently repeated over one or three times with similar results as stated in the Figure Legends. Most of attempt at replication were successful.
Randomization	For cell experiments, all cells were randomly allocated from the same parental cells and treated with the same indicated methods. For all animal studies, animals in each group of the same gender, age, and genetic background were randomized.
Blinding	Animal grouping and immunohistochemical staining analysis were blinded.The data of in vitro and in vivo were processed and analysis by the blinded co-author mentioned in Materials and Methods.

Reporting for specific materials, systems and methods

We require information from authors about some types of materials, experimental systems and methods used in many studies. Here, indicate whether each material, system or method listed is relevant to your study. If you are not sure if a list item applies to your research, read the appropriate section before selecting a response.

Materials & experimental systems

n/a	Involved in the study	
☐	☒	Antibodies
☐	☒	Eukaryotic cell lines
☒	☐	Palaeontology and archaeology
☐	☒	Animals and other organisms
☐	☒	Human research participants
☐	☒	Clinical data
☒	☐	Dual use research of concern

Methods

n/a	Involved in the study	
☒	☐	ChIP-seq
☐	☒	Flow cytometry
☒	☐	MRI-based neuroimaging

Antibodies

Antibodies used	For flow cytometry, the antibodies used included anti-AXL-PE (Thermo Fisher Scientific, #12-1087-42, clone DS7HAXL), anti-human CD3-FITC (Biolegend, #300406, clone UCHT1), anti-human CD4-APC (Biolegend, #300514, clone RPA-T4), anti-human CD8a-PE (Biolegend, #300908 clone HIT8a), anti-human TIM-3-PE (BD, #563422, clone 7D3), anti-human CTLA-4 PercP-Cy5.5 (Biolegend, #369607 clone BNI3), anti-human PD1-APC (Biolegend, #329908, clone EH12.2H7), anti-human CD95-APC (Biolegend, 305612, clone DX2), anti-human CD25-PE (Biolegend, #985802 clone M-A251), anti-human CD3-APC-CY7 (Biolegend, #317342 clone OKT3), anti-human CD4-PerCP (Biolegend, #300528, clone RPA-T4), anti-human CD8-PE-CY5 (Biolegend, #344769, clone SK1), anti-human CD25-APC (Biolegend, #302609, clone BC96), anti-human CD69-APC-CY7 (Biolegend, #310913, clone FN50), anti-human FOXP3-PE

	(Biolegend, #320107 clone 206D), anti-human CD45RO-PerCP/Cy5.5 (Biolegend, #304222, clone UCHL1), anti-human CD62L-PE (Biolegend, #304806, clone DREG-56), anti-human CD68-APC (Biolegend, #333810, clone Y1/82A), anti-human CD19-PE (Biolegend, #302208 clone HIB19), anti-human CD14-PE-CY7 (Biolegend, #301814, clone M5E2), anti-human CD56-APC-CY7 (Biolegend, #362512 clone 5.1H11), anti-human CD206-PE (Biolegend, #321106, clone 15-2), mouse IgG1 kappa isotype control-PE (Biolegend, #400112, clone MOPC-21), mouse IgG1 kappa isotype control-FITC (Biolegend, #400108, clone MOPC-21), mouse IgG1 kappa isotype control-APC (Biolegend, #400120, clone MOPC-21), mouse IgG2a kappa isotype control-PerCP/Cy5.5 (Biolegend, #400252, clone MOPC-173), mouse IgG2a kappa isotype control-APC-CY7 (Biolegend, #400230, clone MPC-173), mouse IgG1 kappa isotype control-PerCP (Biolegend, #400148, clone MOPC-21), mouse IgG1 kappa isotype control-PE-CY5 (Biolegend, #400118, clone MOPC-21), mouse IgG2b kappa isotype control-APC (Biolegend, #400322, clone MPC-11), mouse IgG2a kappa isotype control-PE-CY7 (Biolegend, #400232, clone MOPC-173), mouse IgG1 kappa isotype control-APC-CY7 (Biolegend, #400128, clone MOPC-21).<br><br>Primary antibodies for WB and IHC, including rabbit anti-AXL antibody (1:500, Cell Signalling Technology, #8661, clone C89E7), anti-human CD3 (1:25, Abcam, ab16669, clone SP7), C1QBP (1:1000, Cell Signaling Technology, #6502, clone D7H12); human CD31 (1:1000, Cell Signaling Technology, #3528, clone 89C2), GFP (1:800, ab290, Abcam), C1QBP (1:1600, Cell Signaling Technology, #6502, clone D7H12), HLA-A (1:100, Abcam, ab52922, clone EP1395Y) and Ki-67 (1:500, Abcam, ab231172, clone SP6)
Validation	All antibodies, like Abcam or CST, are commercially available and have been tested for species reactivity and validated by the manufacturers and supplier. The statements of validation of each primary antibody for the species and application are avilable on the manufacturer's websit, like rabbit anti-AXL antibody (1:500, Cell Signalling Technology, #8661, clone C89E7), website link: https://www.cellsignal.cn/products/primary-antibodies/axl-c89e7-rabbit-mab/8661?site-search-type=Products&N=4294956287&Ntt=8661&fromPage=plp&_requestid=7858697.

Eukaryotic cell lines

Policy information about cell lines

Cell line source(s)	A549, HCC827, and HEK-293T cell lines were obtained from the American Type Culture Collection. The erlotinib-resistant HCC827-ER3 cell line was established at the Case Western Reserve University (Cleveland, Ohio, USA) as previously described. The Free-Style 293-F cells were obtained from Invitrogen and cultured in Freestyle 293 expression medium (Gibco). Cells were maintained in a humidified atmosphere of 95% air and 5% CO2 at 37 °C in DMEM (Gibco) or RPMI-1640 (Gibco) supplemented with 10% foetal bovine serum (Gibco), 100 IU/mL penicillin (Gibco), and 100 IU/mL streptomycin (Gibco).
Authentication	All cell lines were authenticated by short tandem repeat (STR)-profiling.
Mycoplasma contamination	All cell lines were tested negative for mycoplasma contamination.
Commonly misidentified lines (See ICLAC register)	No misidentified cell lines were used in this study.

Animals and other organisms

Policy information about studies involving animals; ARRIVE guidelines recommended for reporting animal research

Laboratory animals	Six- to eight-week-old female NSG (NOD-PrkdcscidIl2rgtm1/Bcgen, Biocytogen, Beijing, China) mice and the fourteen to sisteen-week-old female humanized NOG-EXL mice (NOD.Cg-Prkdc scid Il2rg tm1Sug Tg(SV40/HTLV-IL3,CSF2)10-7Jic/JicTac) expressing human GM-CSF and human IL-3, engrafted with CD34+ human cord blood stem cells (Taconic Biosciences) were housed under specific pathogen-free conditions at a constant temperature (22 ± 0.5 °C) and humidity (60 ± 2%) under an automatically controlled 12/12 h light/dark cycle, and were provided autoclaved food and water at the Experimental Animal Centre of the Second Affiliated Hospital of Guangzhou Medical University (Guangzhou, China).
Wild animals	Wild animals were not involved in this study.
Field-collected samples	Field -collected samples were not involved in this study.
Ethics oversight	All human sample collections and analysis were approved by the Institutional Review Board of the Second Affiliated Hospital of the Guangzhou Medical University. Animal experiments were performed with the approval of the Second Affiliated Hospital of Guangzhou Medical University Experimental Animal Care Commission.

Note that full information on the approval of the study protocol must also be provided in the manuscript.

Human research participants

Policy information about studies involving human research participants

Population characteristics	1.To detect AXL expression profile, human NSCLC and normal tissues were collected and detailed in Table S1-3.<br>2.To establish patient-derived xenografts model, NSCLC tumour specimens were collected from one patient at the Second Affiliated Hospital of Guangzhou Medical University (Guangzhou, China).
Recruitment	In order to collect human peripheral T cells, healthy donors from our institution were recruited in this study on the basis of willingness, and three donors (2 male, 1 female, mean age, 25-year-old) without organic or systemic diseases were selected.

Ethics oversight	Institutional Review Board (IRB) of the Second Affiliated Hospital of Guangzhou Medical University (Guangzhou, China) was obtained. The informed consent was obtained by human participants.

Note that full information on the approval of the study protocol must also be provided in the manuscript.

Clinical data

Policy information about clinical studies
All manuscripts should comply with the ICMJEguidelines for publication of clinical research and a completedCONSORT checklist must be included with all submissions.

Clinical trial registration	NA.
Study protocol	NA.
Data collection	Clinical data was collected from medical history with the patient's permission and approved by Institutional Review Board of the Second Affiliated Hospital of Guangzhou Medical University (Guangzhou, China).
Outcomes	NA.

Flow Cytometry

Plots

Confirm that:

- [x] The axis labels state the marker and fluorochrome used (e.g. CD4-FITC).
- [x] The axis scales are clearly visible. Include numbers along axes only for bottom left plot of group (a 'group' is an analysis of identical markers).
- [x] All plots are contour plots with outliers or pseudocolor plots.
- [x] A numerical value for number of cells or percentage (with statistics) is provided.

Methodology

Sample preparation	Cells were centrifuged at 1,000 rpm for 5 min and washed with cold PBS twice. All FACS-related staining procedures were performed on ice for 30 min, and the cells were then washed with PBS containing 1% foetal bovine serum before cytometry analysis. Peripheral blood and tumour samples from mouse xenografts were treated with red blood cell lysis buffer, and the cells were stained with the corresponding antibodies.
Instrument	All samples were analysed using a NovoCyteTM flow cytometer (ACEA Novocyte D2060R).
Software	All results was analysed by Flowjo V10.
Cell population abundance	Ten thousand cells.
Gating strategy	For multicolor flow, first plot gating for live cells, and second plot for CD3+ or GFP+ cells, then detecting the purpose phenotype.

- [x] Tick this box to confirm that a figure exemplifying the gating strategy is provided in the Supplementary Information.
